# Europinidin Mitigates 3-NPA-Induced Huntington’s Disease Symptoms in Rats: A Comprehensive Analysis of Oxidative Stress, Mitochondrial Enzyme Complex Activity, Pro-Inflammatory Markers and Neurotransmitter Alterations

**DOI:** 10.3390/biomedicines12030625

**Published:** 2024-03-12

**Authors:** Khalid Saad Alharbi

**Affiliations:** Department of Pharmacology and Toxicology, College of Pharmacy, Qassim University, Buraydah 51452, Al-Qassim, Saudi Arabia; khalid.alharbi9@qu.edu.sa

**Keywords:** 3-nitropropionic acid, cognitive dysfunction, europinidin, Huntington’s disease, neurodegenerative diseases, antioxidant

## Abstract

Huntington’s disease (HD) is a neurodegenerative disease that causes progressive motor and cognitive dysfunction. There is no cure for HD, and current therapeutics can only manage the signs and symptoms as well as slowing disease progression. This investigation examines the possible therapeutic advantages of europinidin in 3-nitropropionic acid (3-NPA) injected HD in rats. *Wistar* rats were randomly assigned to five groups (*n* = 6): normal control, 3-NPA (10 mg/kg, i.p.), 3-NPA + europinidin-10 (10 mg/kg, p.o.), 3-NPA + europinidin-20 (20 mg/kg, p.o.), and europinidin alone (20 mg/kg, p.o.) for 15-day. Various behavioral and biochemical parameters including antioxidant levels, oxidative stress, pro-inflammatory markers, mitochondrial enzyme complex, and neurotransmitters were assessed. Europinidin restored biochemical, mitochondrial dysfunction, oxidative stress, neurotransmitter, and pro-inflammatory parameters disrupted by 3-NPA. Here we show that europinidin attenuates 3-NPA-induced neurodegeneration in rat models of HD. Europinidin modulates oxidative stress, enhances antioxidants, restores mitochondrial enzyme complex activity, reduces neuroinflammation, and modulates neurotransmitter levels. Our findings reveal the potential of europinidin as a novel therapeutic agent for the treatment of HD. This study also provides new insights into the molecular mechanisms of europinidin-mediated neuroprotection and may have a beneficial role in the management of neurological diseases.

## 1. Introduction

Background of HD: Huntington’s disease (HD) is a complicated dysfunction which results in damage to nervous system. Neuropathology linked to pronounced motor dysfunctions, like dystonia and ataxia, is the neuropathology that it most frequently exhibits. In this manifest disease, striatal neuronal degeneration results in the loss of function [1,2,3]. According to several studies, the *huntingtin* (HTT) gene’s repeats of the trinucleotides cytosine, adenine, and guanine (CAG) on the shorter chromosomal arm cause HD-like symptoms to manifest. Repeats in the *HTT* gene create mutations that result in an atypical polyglutamine extension, which leads to neurodegeneration [4,5]. The *HTT* protein also aggregates as a result of this enlargement. HD typically manifests between the ages of 30 and 50, but, due to the repetition of chromic nucleotides, earlier onset of the illness is possible. Juvenile HD refers to the early onset of HD in individuals aged 20 or younger, characterized by significant behavioral and learning difficulties. The mutated *HTT* gene in HD leads to the production of a mutant huntingtin protein (mHTT), and the mechanisms by which this mutant protein affects neural substrates and leads to neuronal degeneration are complex. Several processes contribute to the pathogenesis of HD, including protein aggregation, excitotoxicity, mitochondrial dysfunction, and impaired axonal transport [5,6,7].

Throughout the post-embryonic stage, the HTT protein gene reflects essential synaptic neural functions. According to researchers, it prevents apoptosis and guards against harmful mutant HTT. Earlier studies have suggested that the number of mutations in a protein can affect how functional it is. Intranuclear and intracytoplasmic inclusions can be found in several brain regions [1,2,8].

HD is a debilitating neurodegenerative disorder characterized by progressive neuron degeneration in specific brain areas. It results from a mutation in the *HTT* gene, which produces an undesirable protein that disrupts normal cellular processes. Scientists are actively examining a variety of ways to obtain a better understanding of the mechanisms behind this condition and to develop new treatment solutions. One of the key neural substrates affected by HD is the cortico-striatal circuit, which plays a crucial role in motor control and cognitive functions. This circuit involves communication between the cerebral cortex and the striatum, a component of the basal ganglia. The basal ganglia are a group of nuclei deep within the brain involved in motor coordination, as well as aspects of cognition and emotion. To understand its importance in pathophysiology, mitochondrial dysfunction in the emergence of HD is now being investigated. In HD patients who have undergone brain autopsy research, mitochondrial dysfunctions have been found [5,6,7,9].

Role of europinidin: It is possible to treat neurodegenerative illnesses with therapeutic approaches meant to stop neuronal deterioration. The use of natural anti-inflammatory and antioxidant substances found in plants are gaining popularity; these may lessen the danger of cell death and damage brought on by ingesting certain neurotoxins [10,11,12].

The herbaceous species *Ceratostigma plumbaginoides* and *Plumbago europea,* which produce the o-methylated derivative of delphinidin known as europinidin, are members of the *Plumbaginaceae* family. *Plumbago europea*, a perineal herb, is used to treat a diverse range of illnesses including cancer, immunosuppression, dysmenorrhea, hepatitis, respiratory problems, edema, and scabies [13,14,15,16].

3-NPA-induced HD symptoms: HD-like symptoms can be generated experimentally by exposing a particular neurotoxins to animal models. The natural neurotoxin known as 3-NPA is produced by many fungi and plant species, including *A. flavus* and *A. arthrinium*. By blocking mitochondrial complex II, 3-NPA specifically induces GABAergic degeneration in the striatum, which results in rodent neuronal death and is seen in HD patients. Animals treated with 3-NPA display motor behavioral problems, such as abnormal gait, inability to balance over a small beam, deficiencies in exploratory behaviors, and/or elevated levels of anxiety. This model is a useful resource for evaluating the efficacy of potential HD therapeutics because the 3-NPA injection is known to cause HD-like symptoms in animals with a phenotype comparable to the inherited human disease [17,18,19,20,21,22]. Previously, europinidin has been reported as showing a favorable effect against alcohol-induced liver damage [23], streptozotocin-induced memory impairment [24], and rotenone-activated Parkinson’s disease in rats [25].

Objectives of the study: The objective of the study is to investigate the potential anti-Huntington effects of europinidin, a natural anthocyanidin, against 3-NPA, a mitochondrial toxin, in rats. Moreover, the study aims to determine the levels of oxidative stress, mitochondrial enzyme complex, pro-inflammatory markers, neurotransmitters, and cognitive and motor deficits in 3-NPA-induced rats.

## 2. Methodology

### 2.1. Animals

Male *Wistar* (180 ± 20 g; 10–12 weeks old) rats were housed in standard settings, a constant temperature of 24 °C, and 50–60% humidity control. Rats were given a diet that contained 60% fat and had unrestricted access to water. Before being included in the trial, rats were exposed to these conditions in the lab for a period of seven days without receiving any previous medication. The rodent study and tests carried out in accordance with the ARRIVE protocol were authorized by an institutional research board at Qassim University, Saudi Arabia (23-69-13).

### 2.2. Drugs and Chemicals

Europinidin (98.0% purity) and 3-NPA were obtained from MSW Pharma, chandrapour, Maharashtra, India. The enzyme-linked immunosorbent assay (ELISA) kit for rats was utilized to determine neuroinflammatory markers (Krishgen Biosystems, Mumbai, Maharashtra 400018, India).

### 2.3. Acute Toxicity Study

An acute toxicity study was conducted based on OECD guideline no. 423. We monitored the rats for toxicity signs throughout the next 14 days. Europinidin had previously been administered orally at the recommended safe dose [23,24,25]. Several clinical symptoms were observed, including changes in behavior, vision, weight, skin, and fur.

### 2.4. Experimental

The following clusters (*n* = 6) were randomly assigned to a total of 30 rats.

$$\left.\begin{array}{l} I:\;\mathrm{Normal}\\ \mathrm{II}:\;3-\mathrm{NPA}-\mathrm{injected}\;\left(10\;\mathrm{mg}/\mathrm{kg},\;i.p.\right)\\ \mathrm{III}:\;3-\mathrm{NPA}+10\;\mathrm{mg}/\mathrm{kg}-\mathrm{europinidin}\\ \mathrm{IV}:\;3-\mathrm{NPA}+20\;\mathrm{mg}/\mathrm{kg}-\mathrm{europinidin}\\ V:\;20\;\mathrm{mg}/\mathrm{kg}-\mathrm{europinidin}\;\mathrm{per}\;\mathrm{se} \end{array}\right\}\mathrm{Groups}$$

Every day 1 h prior to the above treatments, groups I and III were treated with the normal group, receiving 3 mL/kg of saline solution for 15 days; 10 mg/kg of 3-NP was injected (i.p.) in the morning session. To induce symptoms resembling HD, 3-NPA dispersed in a buffered solution of neutral pH was intraperitoneally administered once daily for 15 days at 10 mg/kg. The dose was chosen based on prior studies [13,14]. An oral dose of europinidin at both 10 and 20 (mg/kg) was given one hour before the 3-NPA treatment. On the last day of the experimental schedule, a behavioral test was conducted on the rats. Rats were anesthetized intraperitoneally with ketamine and xylazine at the end of the experiment and biochemical tests were conducted on the rats’ brains on day 16.

#### 2.4.1. Body Weight

During the randomization procedure and at the end of treatment, animal weights were taken, i.e., before a biochemical estimation on the last day of the treatment.

#### 2.4.2. Grip Strength

An electronic grip force meter was used to gauge the forelimb muscles’ grip strength. This was measured by pulling the rat while it was made to grasp the grid with its forelimbs. The grip force was measured in kilograms [26,27,28].

#### 2.4.3. Rotarod Test

Weekly assessments of rodent integrity, grip strength, and motor synchronization were made using a rotarod apparatus, somewhat modifying previously published results. A non-slippery shaft is divided into four equal pieces to make up the rotarod apparatus. Each animal’s previous training was used to acquaint the rats with the equipment. In both tests, the rotating shaft had a seven cm diameter and rotated at a constant speed of 25 rpm while the rats were being trained. A restricted duration of 180 s was implemented to evaluate the drop-down delay [20,21,22,26].

#### 2.4.4. Balance and Motor Coordination (Beam Walking Test)

The goal of this test was to gauge the animals’ motor coordination and balance. The animals had to move through a narrow, steep tunnel. It was noted how frequently their feet slipped and how long it took them to cross the walkway. Before time ran out, the animals only had 60 s to get across the beam [20,21,22].

#### 2.4.5. Spontaneous Locomotor Activity

On the first and last days of the trial, an actophotometer was used to track locomotor activity. A digital counter displayed an electric impulse produced by each beam interruption on the x- or y-axis. The equipment was put in a testing room that was ventilated, darkened, and sound- and light-attenuated. Values are provided as counts per five minutes for each animal after 5 min of observation [29]. 

### 2.5. Estimation of Biochemical Parameters

#### 2.5.1. Brain Tissue Homogenization

Under light anesthesia, the animals were dislocated on day 16. It had previously been reported that mitochondria could be isolated. Each brain’s striatal tissues were removed, followed by centrifugation of homogenate for 30 min at 4 °C at 5000 rpm. Acute oxidative stress parameters including acetylcholinesterase (AChE), gamma-aminobutyric acid (GABA), and dopamine (DA) levels were estimated from supernatant recovered before recentrifugation (striatal tissues were used to assess all the biochemical parameters). The crude mitochondrial pellet was separated following recentrifugation, [18,19,20,21,29].

#### 2.5.2. Estimation of Mitochondrial Complex

A method that has been previously described involved measuring the NADH dehydrogenase activity (mitochondrial complex-I) by catalyzing the oxidation of NADH and then reducing cytochrome c. Absorbance at 550 nm was recorded. The findings were presented in terms of nmol NADH oxidized/min/mg protein [29].

Using the technique described in a previous article, the activity of SDH (mitochondrial complex-II) was evaluated in the mitochondrial fraction. Absorbance was measured at 420 nm. The results were expressed in terms of nmol succinate oxidized/min/mg protein [29].

The analysis for cytochrome oxidase (mitochondrial complex-IV) was carried out in the mitochondrial fraction using the procedure developed by Sottocasa et al. The decrease in absorbance was recorded at 550 nm. As a measure of cytochrome c oxidation, the molar extinction coefficient of cytochrome c was used [28,30,31,32].

#### 2.5.3. Detection of AChE

The mixture comprised Acetylcholine Iodide (1 mM), DTNB (2 mM), potassium phosphate buffer (100 mM) at pH 7, and hippocampus/striatum homogenate, with a total volume of 500 L. Incubation was conducted at 37 °C for 10 min. To terminate the reaction, serine hemisulfate (0.5 mM) was added. A yellow-colored solution was obtained, and it was analyzed at 412 nm to determine the concentration of AChE.

#### 2.5.4. Determination of GABA

Neurotransmitter concentrations, including GABA was assessed using by high-performance liquid chromatography (HPLC Agilent (1100), Agilent Technologies, Waldbronn, Germany). The preserved brain samples were thawed and blended in 0.2 M perchloric acid. The samples were centrifuged at 12,000× *g* for 15 min. Supernatants were separated. Supernatant was passed through 0.22 mm nylon filters before being injected into the HPLC sample injector [31].

#### 2.5.5. Estimation of DA and Glutamate

The previously described approach was followed for the measurement of dopamine levels in the striatum. Striata were weighed and homogenized in 0.1 M perchloric acid containing 0.05% EDTA for 10 s. The samples came from the right hemisphere of the brain. The supernatant from homogenate was subjected to high-pressure chromatography [29,31,32,33]. The amounts of glutamate were determined as per the standard procedure.

#### 2.5.6. Estimation of SOD

To evaluate superoxide dismutase (SOD) activity, nitroblue tetrazolium (NBT) was photochemically degraded by SOD. The activity was determined as per the previously reported protocol. SOD activity was quantified in units per milligram (U/mg) [34,35,36,37,38].

#### 2.5.7. Estimation of Reduced Glutathione (GSH)

Tissue homogenates were processed as described in past processes, and solutions obtained from the supernatant were measured at 412 nm in comparison to a blank after the entire reaction was complete. The concentration was calculated in U/mg [28,30,31,32,33].

#### 2.5.8. CAT Measurement

After the processing of homogenate, the supernatant was isolated. A spectrophotometer operating at a wavelength of 570 nm was used to measure the CAT. Additionally, parallel processing was carried out with tubes devoid of any enzyme. The catalase activity in U/mg was calculated using the variation in absorbance per unit [31,32].

#### 2.5.9. Determination of MDA

Test tubes were filled with 500 mL of homogenate, while the control tube received 500 µL of Tris-HCl buffer (50 mM, pH 7.4). Totals of 500 µL of 0.67% TBA and 250 mL of 20% trichloroacetic acid (TCA) were added to each tube. The tubes were sealed with glass beads and subjected to incubation in a water bath at 90 °C for 10 min. After cooling to room temperature, the tubes were centrifuged for 15 min at 3000 rpm. The absorbance of the supernatant at 530 nm was calculated, with the control serving as a reference. MDA concentrations were calculated as (nmol/L) [31,32].

#### 2.5.10. Neuroinflammatory Markers

Neuroinflammatory cytokines are known to be elevated in the brains of subjects with HD, and they contribute to neuronal damage and neurodegeneration. Using conventional ELISA kits and following established protocols, IL-1β, IL-6, and TNF-α were quantified. The quantities were calculated as pg/mL [31,32].

As far as the level of BDNF concerned, neurotrophic factors are essential for neuronal survival, growth, and differentiation. HD is associated with reduced levels of BDNF, and therapies that promote BDNF expression may prove effective. The obtained brain homogenate was exposed to BDNF antibodies. Levels of BDNF were assessed using common assay kits [31,32].

#### 2.5.11. Statistical Analysis

The mean ± 95% confidence intervals (CI) were used to calculate the outcome. Following a one-way analysis of variance (ANOVA), a Tukey’s post hoc test was undertaken and achieved a significance level of *p* < 0.05. Following an ANOVA, the validity of the data was confirmed using Shapiro–Wilk normality tests.

## 3. Results

### 3.1. Acute Toxicity Study

No mortality or any other abnormality was observed during the acute toxicity period. We used europinidin at 10 and 20 mg/kg based on the acute toxicity study (Table 1).

### 3.2. Body Weight

**Figure 1:**
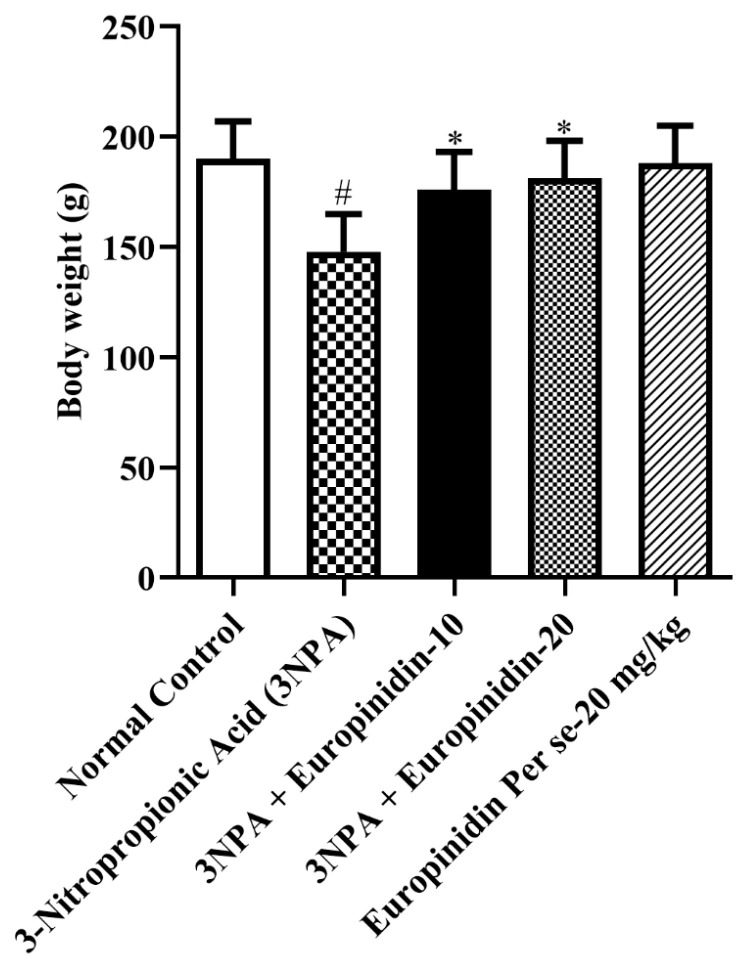
The effect of europinidin on body weight. Mean ± 95% CI. (*n* = 6). # *p* < 0.0001 vs. Normal control, * *p* < 0.05 vs. 3-NPA control. One-way ANOVA following Tukey’s test.

As shown in Figure 1, it was observed that the body weight of 3-NPA-injected rats was reduced by a substantial extent *p* < 0.01 in association with normal. Europinidin increased the body weight of rats to a significant extent [F (4, 25) = 6.564, (*p* = 0.0009)]. No significant effect was observed in the europinidin per se cluster.

### 3.3. Grip Strength

**Figure 2:**
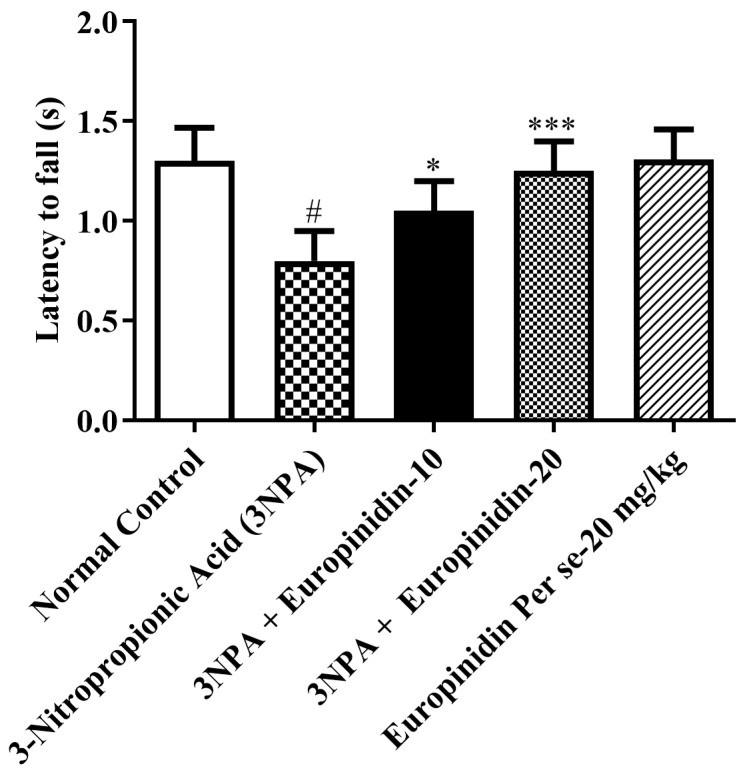
The effect of europinidin on grip strength. Mean ± 95% CI. (*n* = 6). # *p* < 0.0001 vs. Normal control, * *p* < 0.05, *** *p* < 0.0001 vs. 3-NPA control. One-way ANOVA following Tukey’s test.

As presented in Figure 2, the 3-NPA-injected rats exhibited significantly decreased grip strength as compared to normal. The europinidin in both treatments resulted in significant [F (4, 25) = 13.37, (*p* < 0.0001)] enhancement of grip strength to a substantial extent as compared to the 3-NPA-injected rats. The europinidin per se group showed no effect as compared to the normal group. This suggests that europinidin has a significant positive impact on improving grip strength in the context of 3-NPA-induced reduction.

### 3.4. Rotarod Test

**Figure 3:**
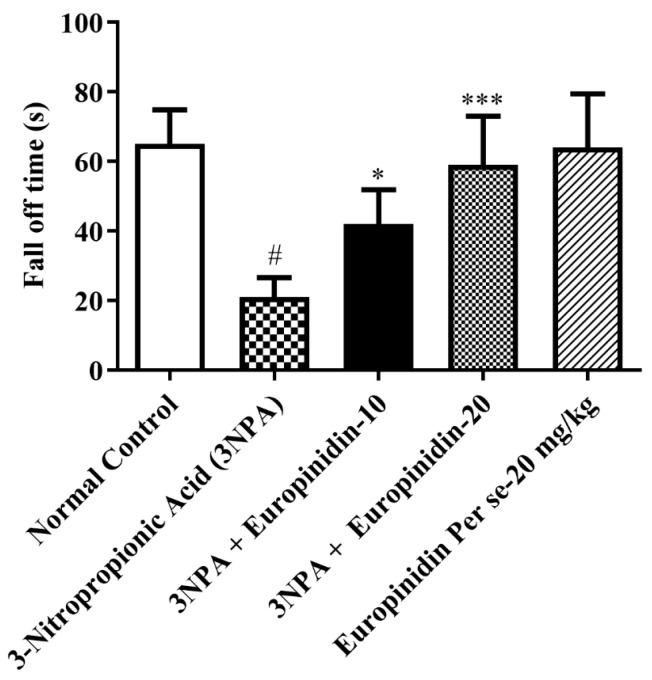
The effect of europinidin on the rotarod test. Mean ± 95% CI. (*n* = 6). # *p* < 0.0001 vs. Normal control, * *p* < 0.05, *** *p* < 0.0001 vs. 3-NPA control. One-way ANOVA following Tukey’s test.

It was observed that 3-NPA-injected rats displayed a substantial decrease in latency to fall [F (4, 25) = 17.54, (*p* < 0.0001)] compared to normal. Treatment with europinidin at both doses exerted a substantial increase in time to fall (Figure 3). The per se group did not have any associated effect as compared to 3-NPA-injected rats. This suggests that europinidin has a notable impact on improving motor coordination and balance.

### 3.5. Beam Walking Test

**Figure 4:**
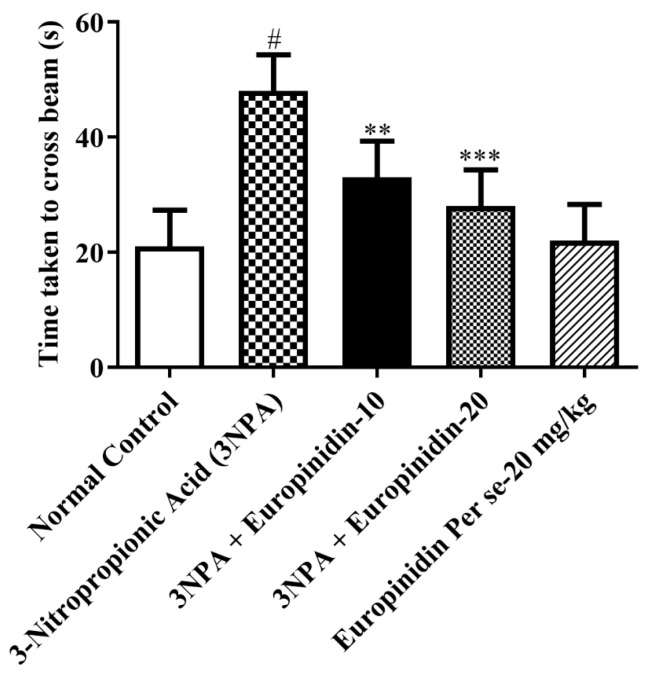
The effect of europinidin on the beam-walk test. Mean ± 95% CI. (*n* = 6). # *p* < 0.0001 vs. Normal control, ** *p* < 0.001, *** *p* < 0.0001 vs. 3-NPA control. One-way ANOVA following Tukey’s test.

It was evident from the results that 3-NPA-injected rats displayed a remarkable increase in time to walk *p* < 0.01 in comparison to normal. However, administration of europinidin treatment at both doses (10 and 20 mg/kg) was able to reduce this time by a considerable extent [F (4, 25) = 19.77, (*p* < 0.0001)]. The per se group did not show any effect (Figure 4). These findings suggest that europinidin has the potential to mitigate the motor impairment induced by 3-NPA.

### 3.6. Spontaneous Locomotor Activity

**Figure 5:**
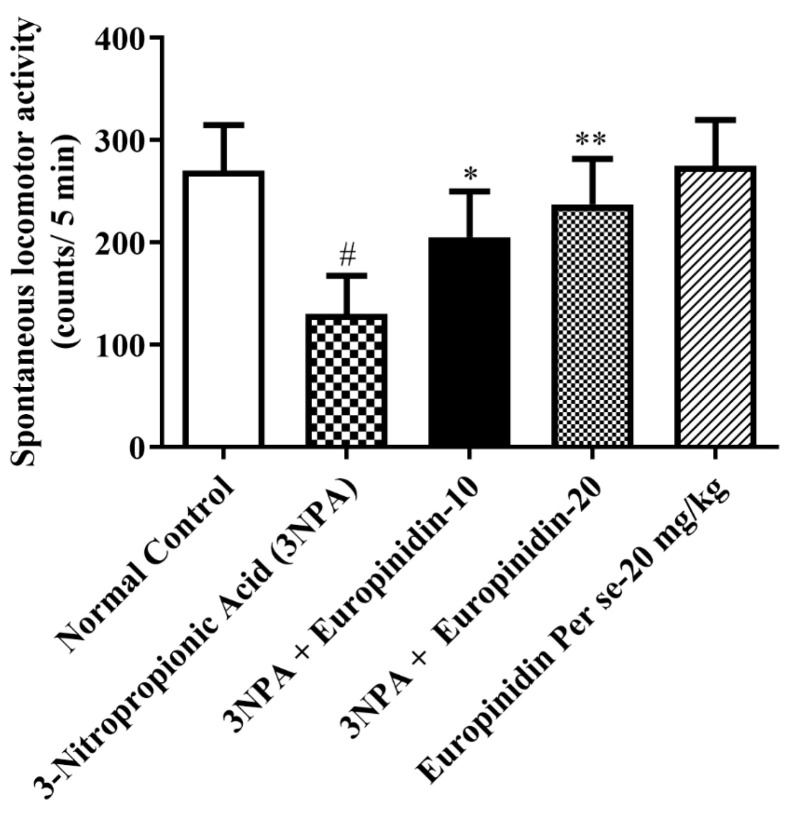
The effect of europinidin on spontaneous locomotor activity. Mean ± 95% CI. (*n* = 6). # *p* < 0.0001 vs. Normal control, * *p* < 0.05, ** *p* < 0.001 vs. 3-NPA control. One-way ANOVA following Tukey’s test.

The 3-NPA treatment significantly reduced the locomotor activity (*p* < 0.01) in comparison to normal. In contrast, treatment with europinidin resulted in a substantial increase in locomotor activity compared to 3-NPA-injected group [F (4, 25) = 12.27, (*p* < 0.0001)]. No noticeable impact on the europinidin per se cluster was observed as compared to the normal group (Figure 5). This suggests a potential behavioral improvement associated with europinidin administration in the context of the experimental model of neurodegeneration.

### 3.7. Impact of Europinidin on Mitochondrial Complexes

**Figure 6:**
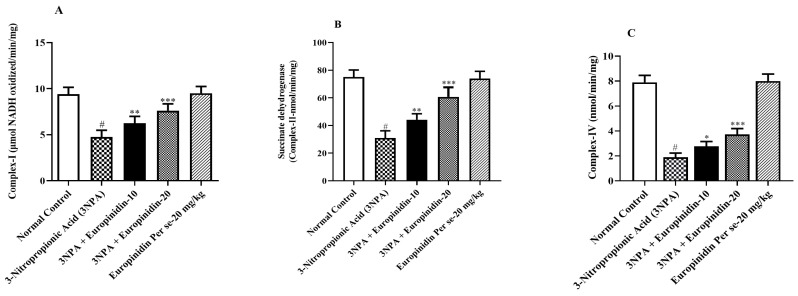
(**A**–**C**): The effect of europinidin on (**A**) Mitochondrial complex-I, (**B**) Mitochondrial complex-II, and (**C**) Mitochondrial complex-IV. Mean ± 95% CI. (*n* = 6). # *p* < 0.0001 vs. Normal control, * *p* < 0.05, ** *p* < 0.001, *** *p* < 0.0001 vs. 3-NPA control. One-way ANOVA following Tukey’s test.

It can be observed from Figure 6A–C that the 3-NPA-injected group showed a noticeable reduction in the amounts of mitochondrial complexes *p* < 0.01 compared to normal. Europinidin significantly elevated the levels of mitochondrial complex-I [F (4, 25) = 50.33, (*p* < 0.0001)], II [F (4, 25) = 82.92, (*p* < 0.0001)], and IV [F (4, 25) = 251.7, (*p* < 0.0001)]. The europinidin per se cluster showed no substantial effect compared to 3-NPA-injected group. These findings suggest that europinidin may play a crucial role in maintaining mitochondrial function.

### 3.8. Effect of Europinidin on AChE

**Figure 7:**
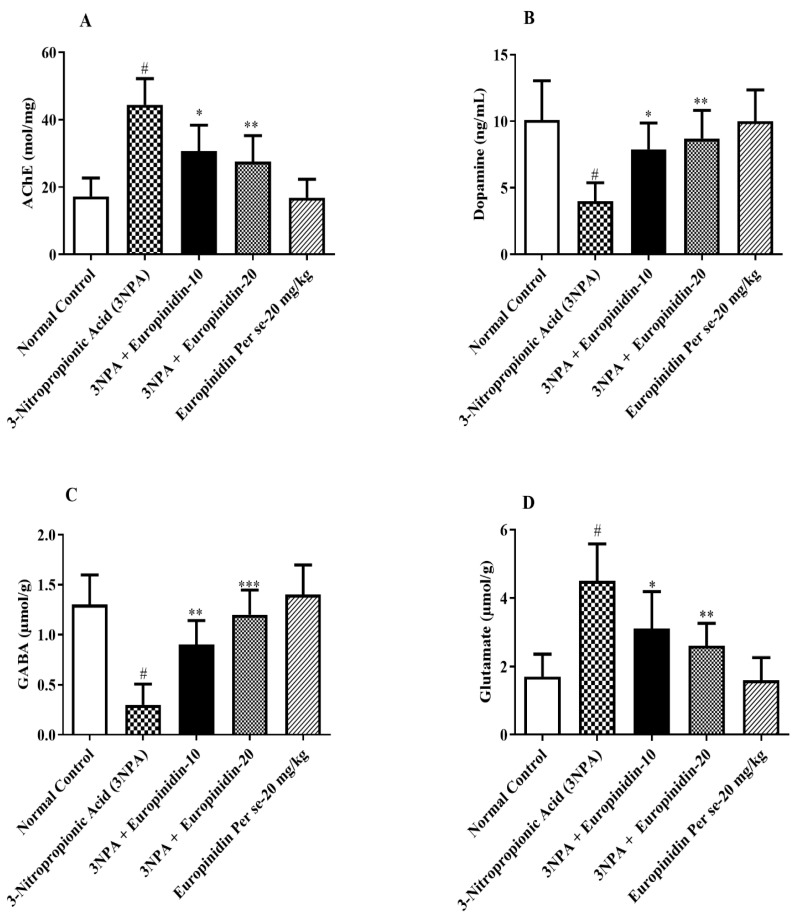
(**A**–**D**): The effect of europinidin on (**A**) AChE, (**B**) Dopamine, (**C**) GABA, and (**D**) Glutamate. Mean ± 95% CI. (*n* = 6). # *p* < 0.0001 vs. Normal control, * *p* < 0.05, ** *p* < 0.001, *** *p* < 0.0001 vs. 3-NPA control. One-way ANOVA following Tukey’s test.

Figure 7A shows that 3-NPA-injected rats displayed significantly increased AChE activity in their brains (*p* < 0.001) compared to normal rats. In brain tissue treated with europinidin, AChE activity was substantially decreased compared to 3-NPA-injected rats [F (4, 25) = 17.77, (*p* < 0.0001)]. This suggests that europinidin has the potential to modulate AChE activity, possibly mitigating the enhanced cholinergic activity. A similar effect was not observed for the europinidin per se group compared to the normal group.

### 3.9. Impact of Europinidin on GABA, DA, and Glutamate

3-NPA-injected rats showed a significant decrease in the amounts of GABA (*p* < 0.01) compared to the normal group. In contrast, treatment with europinidin resulted in a substantial elevation of GABA levels [F (4, 25) = 19.21, (*p* < 0.0001)] compared to 3-NPA-injected rats. This suggests that europinidin reversed the decline in GABA associated with 3-NPA injection.

DA levels were lowered in 3-NPA-injected rats by a considerable extent (*p* < 0.01) compared to the normal group. However, europinidin treatment at both doses (10 and 20 mg/kg) significantly increased DA levels compared to 3-NPA-injected rats [F (4, 25) = 8.369, (*p* = 0.0002)]. It was also observed that the concentration of glutamate was high in 3-NPA-injected rats in comparison with normal. Europinidin treatment at both doses (10 and 20 mg/kg) significantly decreased glutamate levels compared to 3-NPA-injected rats [F (4, 25) = 12.69, (*p* < 0.0001)]. The europinidin per se group showed no effect relative to the normal group (Figure 7B–D). This suggested a potential role for europinidin in mitigating neurochemical alterations.

### 3.10. Oxidative Stress and Antioxidant Markers

**Figure 8:**
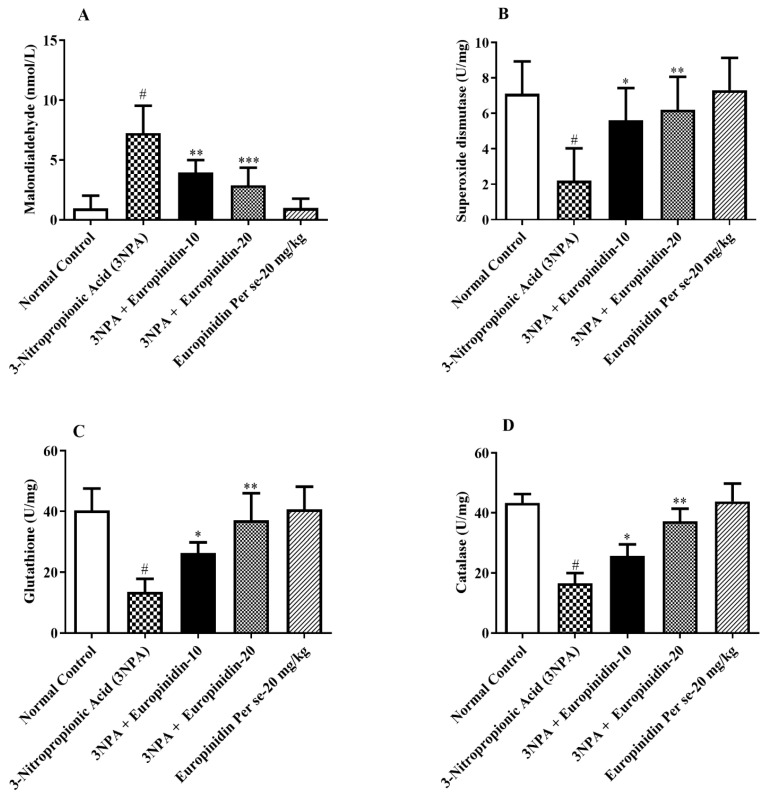
(**A**–**D**): The effect of europinidin on (**A**) MDA, (**B**) SOD, (**C**) GSH, and (**D**) CAT. Mean ± 95% CI. (*n* = 6). # *p* < 0.0001 vs. Normal control, * *p* < 0.05, ** *p* < 0.001, *** *p* < 0.0001 vs. 3-NPA control. One-way ANOVA following Tukey’s test.

The effect of europinidin on MDA levels in 3-NPA-injected rats was evaluated using a one-way ANOVA. MDA was increased by a considerable amount in the 3-NPA-injected rats compared to normal rats (*p* < 0.001). Post hoc tests estimated a remarkable reduction in MDA levels with europinidin at both doses (10 and 20 mg/kg) [F (4, 25) = 21.87, (*p* < 0.0001)]. Furthermore, the europinidin per se group showed no effect compared to the normal group. (Figure 8A)

As presented in Figure 8B–D, post hoc tests revealed that 3-NPA-injected rats had a notable reduction in SOD, GSH, and CAT activity (*p* < 0.001) in comparison to normal rats. These declining enzymes were significantly increased in 3-NPA-injected rats after receiving the europinidin treatment at both doses (10 and 20 mg/kg) [F (4, 25) = 8.332, (*p* = 0.0002)], [F (4, 25) = 20.42, (*p* < 0.0001)], and [F (4, 25) = 52.48, (*p* < 0.0001)] compared to the 3-NPA-injected rats group. There was no effect observed in the europinidin per se group.

### 3.11. Neuroinflammatory Indicators

**Figure 9:**
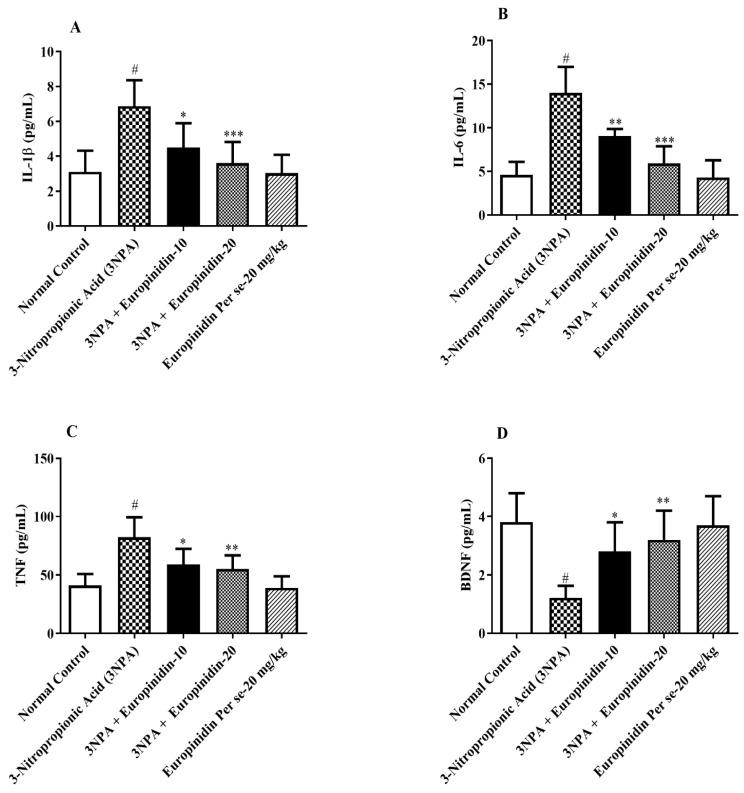
(**A**–**D**): The effect of europinidin on (**A**) IL-1β, (**B**) IL-6, (**C**) TNF-α, and (**D**) BDNF. Mean ± 95% CI. (*n* = 6). # *p* < 0.0001 vs. Normal control, * *p* < 0.05, ** *p* < 0.001, *** *p* < 0.0001 vs. 3-NPA control. One-way ANOVA following Tukey’s test.

There was a notable elevation in the concentrations of IL-1β, IL-6, and TNF-α (*p* < 0.001) in 3-NPA-injected rats’ treatment europinidin with restored levels in contrast with normal rats. Treatment with europinidin at doses of 10 and 20 mg/kg reduced the IL-1β, IL-6, and TNF-α levels to a substantial extent [F (4, 25) = 10.13, (*p* < 0.0001)], [F (4, 25) = 27.31, (*p* < 0.0001)], and [F (4, 25) = 12.53, (*p* < 0.0001)] (Figure 9A–C) as compared to 3-NPA-injected rats, indicating a protective effect against neuroinflammation and neurodegeneration. Europinidin per se was not associated with any effect.

### 3.12. BDNF

When normal rats were compared with 3-NPA-injected rats, there was a substantial decrease in the levels of BDNF (*p* < 0.001). Post hoc tests revealed that with europinidin treatment at both doses, the levels of BDNF significantly increased [F (4, 25) = 8.666, (*p* = 0.0002)] compared with 3-NPA-injected rats. No effect was seen in the europinidin per se group (Figure 9D).

## 4. Discussion

A recent study has suggested that 3-NPA significantly compromises a rat’s motor abilities. Rotarod, beam walking, and spontaneous locomotor activity tests were conducted on the animals, which revealed decreased grip strength, motor incoordination and imbalance, decreased motor activity, and increased immobility [1,2,3,8]. In addition, 3-NPA reduced the rat’s discrimination index during an object recognition test, indicating a significant decrease in cognitive performance [39,40,41].

The primary manifestations of HD include deficits in both motor function and cognition. The changes observed in rodents following the administration of 3-NPA, such as motor impairment and memory decline, may potentially mirror the symptoms observed in HD patients. Among chemical models for HD in animals, 3-NPA most commonly replicates the clinical signs of HD in experimental subjects. Although the pathophysiology of HD is not yet fully understood, investigations into 3-NPA-induced neurotoxicity have provided insights into its pathology, highlighting the significant roles of oxidative stress and mitochondrial dysfunction [4,5,6,7]. In this study, the researchers used a minimum dose of 3-NPA to avoid the mortality rate which was previously observed [9,31]. Consequently, it has been observed that pharmacological inhibitors targeting mitochondrial complex II, such as 3-NPA, induce atrial degeneration and motor abnormalities in animals, closely resembling the symptoms observed in individuals with HD. In the rat brain, manifestations of mitochondrial dysfunction, oxidative stress, and memory loss symptoms seen in HD patients [9,10,11,29].

Despite maintaining normal or even increased energy consumption, HD patients frequently experience gradual weight loss. A decline in body weight can serve as an indicator of 3-NPA neurotoxicity, signaling a deceleration in energy metabolism. Supporting the aforementioned finding, during this study’s investigation, 3-NPA-treated rats exhibited drops in body weight and noticeably reduced intake of food. These drops were significantly reversed by europinidin pre-treatment for 14 days, indicating therapeutic potential [12,13,14,42,43].

Motor coordination was shown to be severely decreased by continuous treatment with 3-NPA. In addition to causing a worsening of beam-walking performance and impairing locomotor function, treatment with 3-NPA decreased the animal’s grip strength. On the other hand, it was discovered that europinidin significantly reduced the memory and motor impairments caused by 3-NPA intoxication. The researchers applied four behavioral tests, i.e., grip strength, rotarod test, beam-walking test, and spontaneous locomotor activity, which are the tests most frequently used to measure the extent of HD-like symptoms in animal models in previous studies [2,9]. This paradigm demonstrates high face and predictive validity in the HD model. In this pre-clinical experimental study, it has been discovered that effective control of the mitochondrial signaling pathway is helpful in restoring cyclic nucleotide pathways for preventing cases of HD. Mitochondrial dysfunction is well-documented and investigated in HD. Additionally, improving mitochondrial activity is sufficient to treat rats’ memory and motor impairments [2,3,4,5,6]. The selective and dominant effect of europinidin in the subjects’ brains may be because of its effectiveness in addressing mitochondrial dysfunction. Europinidin may protect mitochondrial integrity by maintaining the activity of mitochondrial enzyme complexes, particularly complexes I, II, and IV. This preservation of mitochondrial function could help sustain cellular energy production and minimize oxidative damage [15,16,17,18,19].

Memory and cognitive problems are mostly brought about by an imbalance of several neurotransmitters in the cholinergic nerve system in HD. Numerous neurodegenerative disorders are connected to the loss of cholinergic innervations, which is shown by higher AChE enzyme levels. Acetylcholine is depleted by increased AChE while acetylcholine availability is prolonged by inhibition of AChE, which improves cholinergic function. This investigation confirmed that 3-NPA treatment, which has been widely documented in numerous research papers, dramatically elevated AChE levels in the context of the group receiving 3-NPA treatment in the current investigation; meanwhile, the test medication substantially decreased the AChE level [7,9,29].

Past research has shown that neurotransmitter levels drop as a result of chemically induced neurotoxicity. Furthermore, important candidates in the pathogenesis of HD have been identified for dopamine, GABA, and glutamate. Additionally, this study has revealed that the infusion of 3-NPA substantially modified the concentration of GABA, DA, and glutamate. Europinidin might influence neurotransmitter levels by inhibiting AChE and glutamate levels, increasing GABA levels, and promoting DA synthesis or release. These actions could help restore neurotransmitter balance, which is often disrupted in HD [34,35,36,37,44].

Abnormal reactive oxygen species (ROS) production, linked to mitochondrial activity disruption inhibition of enzymes in the Electron Transport Chain (ETC), induces oxidative stress, leading to elevated electron leakage from the mitochondria and the synthesis of ROS, including the superoxide radical (O_2_-), hydrogen peroxide (H_2_O_2_), (NO-), and the hydroxyl radical (OH-). Europinidin might influence neurotransmitter levels by inhibiting AChE activity, increasing GABA levels, and promoting DA synthesis or release. These actions could help restore neurotransmitter balance, which is often disrupted in HD [45,46,47,48,49,50].

Earlier investigations, along with the evident deductions from the present study’s data, highlight a significant augmentation of oxidative stress upon 3-NPA administration. This is manifested by heightened production of ROS, increased nitrite levels, elevated products of lipid peroxidation, and declined GSH concentration in rats subjected to 3-NPA. Over a 15-day oral administration period, europinidin exhibited a remarkable dose-dependent reversal of these parameters, prominently diminishing ROS production in 3-NPA-treated rats. The intrinsic defense mechanism against ROS, catalase, showed decreased activity in animals treated with 3-NPA compared to those treated with a vehicle. In contrast, pre-administration of europinidin to rats administered with 3-NPA for 14 days resulted in a noteworthy elevation in CAT levels. Known for its antioxidant properties, europinidin holds the potential to scavenge free radicals and mitigate oxidative stress. This action may involve the enhancement of crucial antioxidant enzymes, such as SOD, GSH, and CAT, contributing to ROS accumulation, including MDA [51,52,53,54,55,56].

Previous investigations have shown that pro-inflammatory markers significantly increased in neurotoxicity. In the course of the current research, it was found that the concentrations of each of the aforementioned indicators were greatly altered by 3-NPA, which was reduced by europinidin. Europinidin may exert anti-inflammatory effects by modulating pro-inflammatory markers. By reducing the levels of these cytokines, it could attenuate neuroinflammation, which is known to contribute to HD progression [33,51,52,53,57,58,59,60].

Previous research has shown that HD experimental rats have reduced BDNF and nitrite levels. Additionally, this decrease is explained by increased ROS generation, downregulated BDNF mRNA, and altered huntingtin protein expression. It has also been found that 3-NPA treatment significantly alters BDNF levels in animals. Additionally, europinidin effectively reverses the restored nitrite and boosts levels of BDNF in rats with 3-NPA-induced neurotoxicity, illustrating a neuro-defensive effect [19,20,21,22,26,27,28,30,31,32]. The per se group did not show any significant effect, indicating that europinidin may be safe and effective in HD-like conditions.

Europinidin, a natural anthocyanidin, is well known for its antioxidant properties [23,24,25]. Antioxidants neutralize ROS and protect cellular components from oxidative damage. Their ability to reduce oxidative stress, inhibit inflammatory signaling, and preserve mitochondrial function contributes to a protective effect against inflammation, mitochondrial dysfunction, and neurotransmitter changes. Europinidin’s potent antioxidant activity may have contributed to the observed biochemical changes, including a reduction in oxidative stress markers. It is hypothesized that europinidin enhances the activity and expression of endogenous antioxidant enzymes, which further reduce oxidative damage to the neurons. Europinidin inhibits AChE and increases the availability of ACh in the synaptic cleft, enhancing the cholinergic transmission and cognitive functions. Furthermore, europinidin restores the balance of these neurotransmitters by increasing the levels of DA and GABA and decreasing the levels of glutamate, which may improve motor function, neuronal stability, and synaptic plasticity. Europinidin inhibits the activation of pro-inflammatory pathways, such as NF-κB, which may be triggered by 3-NPA-induced oxidative stress and mitochondrial dysfunction. Europinidin also suppresses the production and release of pro-inflammatory cytokines, which exacerbate the neuroinflammation and neuronal death in the striatum. Europinidin increases the levels of BDNF in the striatum, promoting neuronal survival and regeneration. Europinidin’s therapeutic effects in the 3NPA-instigated HD rat model are achieved through a combination of antioxidative, mitochondrial-protective, anti-inflammatory, and neurotransmitter-modulating mechanisms. Further research is needed to validate and refine this hypothetical mechanism, shedding more light on europinidin’s potential as a treatment option for HD.

Study limitations include the fact that europinidin was only administered for 15 days to rats with 3-NPA-induced HD symptoms. Additionally, it is important to compare the drug’s efficacy to other available treatment options to determine its effectiveness further. To understand the HD model’s impact on neural injury, specific gene expression, proteins, histopathological data, immunofluorescence, and signaling pathways, techniques such as Western blotting should be employed.

## 5. Conclusions

Europinidin appears to have promising therapeutic effects, according to recent extensive analysis of 3-NPA-injected HD. Europinidin may produce neuroprotective effects via reduced HD symptoms, i.e., reduction of oxidative stress, restoration of mitochondrial enzyme complex activity, inhibition of pro-inflammatory markers, and normalization of neurotransmitter alterations. To fully understand the underlying molecular pathways and to apply these discoveries to new therapeutic approaches for people with HD, additional research is required. Despite these promising results, it is essential to acknowledge study limitations, including the relatively short duration of europinidin administration and the need for further research to validate and refine the proposed mechanisms. Additionally, comparisons with other available treatment options will be crucial to assess europinidin relative efficacy in HD-like conditions. In conclusion, europinidin is a potential therapeutic agent for Huntington’s disease, warranting continued investigation to fully understand its clinical implications and potential for translation into effective treatments for HD patients.

## Figures and Tables

**Table 1:** Acute oral toxicity of europinidin.

Behavioral Changes	Results
Skin and Fur	Normal
Respiration	Normal
Eyes	Normal
Sleep	Normal
Salivation	None
Convulsions	None
Diarrhea	None
Lethargy	None
Coma	None
Mortality	None

## Data Availability

Data are contained within the article.
